# Development and accelerated shelf-life assessment of coconut squash: A comprehensive evaluation of physicochemical, antioxidant, and sensory attributes

**DOI:** 10.1016/j.fochx.2025.102237

**Published:** 2025-01-26

**Authors:** Sara Shahbaz, Iahtisham-Ul-Haq, Nirmeen Nadeem, Mahnoor Siddiqui, Robert Mugabi, Aanchal Sharma, Tawfiq Alsulami, Gulzar Ahmad Nayik

**Affiliations:** a Kauser Abdulla Malik School of Life Sciences, Forman Christian College (A Chartered University) Lahore, Punjab, Pakistan; b Department of Food Technology and Nutrition, Makerere University, Kampala, Uganda; c University Centre for Research and Development, Chandigarh University, Gharuan, Mohali, Punjab, India; d Department of Food Science and Nutrition, College of Food and Agricultural Sciences, King Saud University, Riyadh 11451, Saudi Arabia; e Marwadi University Research Centre, Department of Microbiology, Marwadi University, Rajkot 360003, Gujarat, India

**Keywords:** Coconut milk, Coconut squash, Accelerated shelf life, Elevated temperature, Q10 method, Organoleptic properties

## Abstract

Value addition in food products is essential to meet changing market trends and lifestyle demands, enhancing both quality and market value. This study aimed to develop and evaluate the accelerated shelf-life of coconut squash. Five formulations with varying coconut milk concentrations (100–500 mL/L) were analyzed over 56 days at room temperature (20 °C). The Q10 method, using acid value as a spoilage factor and temperature as an acceleration factor, was employed for shelf-life prediction. Physicochemical and antioxidant properties were monitored, revealing decreases in pH, total sugars, total phytochemicals, and antioxidant activity, alongside increases in acidity, TSS, and FFA during storage. Sensory evaluation identified T5 as the optimal formulation due to its superior quality attributes and a predicted shelf life of 121 days. These findings highlight T5's potential for commercial applications as a high-quality, functional beverage.

## Highlights

• Formulated and evaluated coconut squash with varying coconut milk concentrations.
• Accelerated shelf-life assessment using the Q10 method with acid value as a spoilage marker.
• Significant changes in physicochemical, antioxidant, and sensory properties during storage.
• T5 formulation showed superior sensory quality and a predicted shelf life of 121 days.
• Coconut squash's has high potential as a plant-based functional beverage.

## 1 Introduction

The world is experiencing a massive change in food consumption patterns to experience a better and healthier lifestyle. As compared to synthetic foods which contain unsafe chemicals and food additives, functional foods are preferred by people due to their nutritional and sensory properties (Sharma et al., 2021). Functional foods have several associated health benefits as they are highly enriched with certain bioactive compounds that improve the immune system of the body and protect it from certain diseases. The concept of functional food first originated in Japan in the year 1984 followed by the USA (Hlaváčová et al., 2022). The change in the physical state of the product by adding certain ingredients to increase its value and economic significance is termed as value addition. This provides a premium quality, safe and sustainable product. (Dalal et al., 2019). Value addition not only improves the quality but also increases the economic and market value of the product (Tóth et al., 2020). Now-a-days consumers are more inclined towards healthier eating patterns due to the high prevalence of diseases. Plant-based foods are becoming more common amongst the masses as compared to the animal-based foods which have high caloric index. In this light, plant-based milk has gained significant importance. Plant-based milk contains a high content of bioactive compounds and lesser allergy-causing components as compared to the milk obtained from animals. Especially people who are lactose intolerant prefer to drink plant-based milk which is easy to digest and has a high nutrient content (Wu et al., 2024).

Coconut (*Cocos nucifera* L.) is a very popular fruit of the Arecaceae family and grows mainly in the tropical regions of the world (Mat et al., 2022). It requires around 24–29 °C of temperature for its healthy development and growth as the palm cannot tolerate extreme temperatures. The soil type also plays a very major role in the development of coconut (Moreno et al., 2020). The composition of coconut is largely dependent on its age and variety (Mat et al., 2022). It provides a variety of different products such as coconut meat, coconut oil, coconut water and coconut milk. Coconut milk, obtained from the fresh grated coconut meat is considered to be a nutritionally rich and healthy beverage making it favorable amongst the people worldwide. Coconut milk is mainly composed of around 38 % fat content. It contains mostly saturated fatty acids (greater than 90 %) which has a major portion (45 to 55 %) of medium-chain fatty acids (Chen et al., 2024). Also, it contains 2.7 % to 6.88 % of carbohydrates, 2.06 % to 3.5 % proteins, 52 % to 74.6 % moisture and 0.64 % to 0.9 % ash content (Sitorus & Lapcharoensuk, 2023). It is a rich source of vitamins, calcium, phosphorus, potassium and essential amino acids. It does not contain lactose and the predominant fat in coconut milk is lauric acid. Lauric acid has a fast metabolic rate in the body and is easy to digest. It helps to prevent several diseases such as arteriosclerosis. The proteins of coconut milk are also easily digested in the body as it is mostly comprised of essential amino acids. The proteins do not cause any allergic reactions in the body. Coconut milk has several associated health benefits due to its anti-viral, anti-microbial, anti-carcinogenic and antibacterial nature. It boosts the immune system of the body, supports bone health and has a major role in mind development (Tulashie et al., 2022). Coconut milk, which is commercially available in liquid and cream form, is used in various cuisines, baking industries and confectionaries around the world (Ahmed et al., 2024). UHT sterilized coconut milk is available commercially that has an extended shelf life and stability as compared to the raw coconut milk (Tiravibulsin et al., 2021). Although coconut milk has several nutritional benefits, it also has certain environmental implications that are needed to be addressed. The major concern is abundant water usage for coconut farming particularly in the tropical regions of the world. Therefore, it is essential to consider the efficiency of water usage in coconut cultivation. Conventional coconut farming is responsible for biodiversity degradation, land deterioration and deforestation. There is dire need to adopt more sustainable farming practices to minimize these environmental impacts which integrate coconut production with other crops to preserve soil health and decrease the carbon footprint. However, extending the shelf life of high-fat plant-based beverages presents various challenges. As plant based milk is highly nutritious, it promotes microbial growth that could deteriorate the quality of the product (Sharma et al., 2024). Coconut milk is unstable due to its highly perishable nature. Due to the high fat content of coconut milk, the processes of lipolysis and rancidity promote its spoilage (Lau et al., 2024). Various heat treatments that are used for shelf-life extension could damage the nutrient content, color and texture, promoting spoilage. Sridhar et al. (2021) describe various controlled preservation techniques that are being employed by the farmers to extend the shelf life of the food products and ensure food safety. The effective preservation methodologies include sterilization, pasteurization, cooking, canning, high-pressure processing, ultrasound, refrigeration or freezing, fermentation and drying. These preservation techniques strictly follow the principles of pH regulation, temperature control, reduced moisture content, effective use of natural preservatives and utilizing good packaging materials to ensure product safety and quality.

The market of functional beverages is experiencing a significant growth in Pakistan to promote healthy lifestyles amongst consumers. Food safety and quality management have evolved the food processing industry through technological advancement and the creation of innovative foods with high consumer demands. Squashes are generally preferred by people over synthetic drinks owing to their natural flavor. The noticeable increase in consumer demand for ready-to-drink beverages requires efficient shelf life estimation methods for the fast and easy commercialization of the products. The Q10 method is a relatively easy approach for the expected shelf life determination of food products with minimum practical work. Squash prepared from coconut milk is considered to be a highly nutritious beverage as the milk obtained from different plant sources is more nutritious as compared to cow milk and may contain some added minerals and vitamins. Coconut drinks are very refreshing due to their pleasant aroma, taste and unique flavor making them an effective source of hydration (Kumar et al., 2021; Wu et al., 2023). In the light of above mentioned facts, this article is based on formulating “Coconut Squash” using an optimized recipe for maintaining the nutritional profile and sensory attributes of the product. The main objectives of this study were to develop coconut squash and its accelerated shelf-life assessment to anticipate product stability.

## 2 Methodology

The current research was conducted at the Kauser Abdulla Malik School of Life Sciences, Forman Christian College, Lahore. The study aimed at the development of coconut squash and its accelerated shelf life assessment through the data obtained by performing various physicochemical and phytochemical tests. The expected shelf life of squash was calculated using the Q10 method. Sensory evaluation was conducted to assess the consumer acceptance of the prepared squash treatments.

### 2.1 Procurement of raw materials

The raw materials used in the preparation of coconut squash were fresh coconuts, sugar, citric acid, salt, coconut essence, sodium benzoate and potassium metabisulphite. Coconut, sugar, salt and coconut essence were purchased from the market. Other chemicals were procured from local vendors.

### 2.2 Extraction of coconut milk

The fresh coconuts were washed properly and broke to open. The white meat was grated and processed in the blender with added water in 1:2. The blended mixture was then strained through a muslin cloth and the milk was squeezed out. The milk extraction procedure was repeated 6 times to collect enough milk for the preparation of the squash. In total, around 1500 mL of coconut milk was extracted from 1000 g of coconut meat.

### 2.3 Preparation of coconut squash

Five treatments of coconut squash were prepared using different quantities of coconut milk i.e. 100, 200, 300, 400 and 500 mL/L. Further, sugar (750 g), salt (1 g), citric acid (5 g), sodium benzoate (0.5 g), potassium metabisulphite (0.5 g) and coconut essence (1 mL) were added in the milk and mixed properly. Then, water was added to make the final volume up to 1 L of each treatment. The treatments were labeled as T1, T2, T3, T4 and T5 (Table 1).

**Table 1:** Treatments plan for coconut squash.

Treatments	Coconut Milk (mL)	Sugar (g)	Salt (g)	Citric Acid (g)	Coconut Essence (mL)	Sodium benzoate (g)	Potassium metabisulphite (g)
T1	100	750	1	5	1	0.5	0.5
T2	200	750	1	5	1	0.5	0.5
T3	300	750	1	5	1	0.5	0.5
T4	400	750	1	5	1	0.5	0.5
T5	500	750	1	5	1	0.5	0.5

### 2.4 Storage of the prepared squash

Clean plastic bottles were filled with the squash treatments and kept at the room temperatures for a period of 56 days to carry out the analysis, fortnightly. For the estimation of accelerated shelf life of squash, the treatments were stored at room temperature (20 °C) and elevated temperature (40 °C), separately to observe the change in acid value.

### 2.5 Physicochemical analysis

#### 2.5.1 pH

The pH of the samples was checked by using a digital pH meter (HI 2211) (Tan & Easa, 2021). The pH meter was calibrated before the analysis. The sample was taken in a small beaker. The probe of the pH meter was washed with distilled water and dried completely. It was then dipped in the sample in such a way that the probe of pH meter did not touch the base of the beaker. The stable values on pH meter were noted at 20 °C.

#### 2.5.2 Acidity

The acidity of the samples was evaluated using the titration method (Coulibaly et al., 2023). For this purpose, 10 mL of the squash sample was taken in a beaker and 3–4 drops of phenolphthalein indicator (1 % made in ethanol) were added into it. It was titrated against 0.1 N NaOH solution until persistent light pink color appeared. The volume used for titration was noted. The readings were taken in triplicates and the calculations were carried out using the formula:

(1) $$\text{Acidity}\;\left(\%\right)=\left[\frac{N\times V\times \mathrm{Eq}.\mathrm{wt}}{W\times 1000}\right]\times 100$$

where,

N = Normality of NaOH.

V = Volume of the titrant (mL).

Eq. wt = Weight of citric acid (64.4 g).

W = Weight of the sample (g).

#### 2.5.3 Total soluble solids (TSS)

The brix of the samples was checked using a digital refractometer (Model: LH-T80) (Asghar et al., 2020). A drop of the sample was placed on the lens of the refractometer using a dropper and the reading was recorded.

#### 2.5.4 Free fatty acids (FFA)

The free fatty acid content (% Lauric acid) in the samples was estimated using the titration method (Manoj & Shanmugasundaram, 2020). 2 mL sample was taken in a flask and 20 mL of the freshly neutralized alcohol was added into it followed by the addition of 3–4 drops of phenolphthalein indicator (1 % made in ethanol). The solution was mixed and titrated against the 0.1 N KOH solution. The end point was indicated by the appearance of persistent pink color. The initial and final readings of the burette were noted. Free fatty acid content was quantified using the formula:

(2) $$\text{Free Fatty Acid}\;\left(\%\right)=\frac{N\times V\times 200.32}{W\times 1000}\times 100$$

where,

N = Normality of KOH.

V = Volume of the titrant (mL).

W = Weight of the sample (g).

### 2.6 Total sugars

The total sugars (reducing and non-reducing) in the samples were quantified using the method of Hernández-López et al. (2020). According to the method, 1 mL of each sample was taken and 25 mL distilled water was added into it. The diluted samples were centrifuged at 4000 rpm for 20 min. After centrifugation, 200 μL of the samples were taken in test tubes followed by the addition of 800 μL of distilled mater. 2 mL of Benedict's reagent was then added in all the test tubes. The blank was prepared in the same way by replacing the sample with water. The test tubes were then placed in the water bath for 5 min at 100 °C. The absorbance of all the reaction mixtures was checked at 740 nm using spectrophotometer. The standards of glucose, sucrose, maltose and fructose were also prepared to plot the standard curve and quantify the sugars.

### 2.7 Color analysis

The color analysis of the samples was done using a CIE Lab Color Meter (Navya et al., 2020) by recording the values of L (lightness), a* (+redness and –greenness) and b* (+yellowness and –blueness) parameters. The obtained values of L, a* and b* were used to calculate the chroma and hue angle using the following formulas:

(3) $$\text{Chroma}=\sqrt{{\left(a*\right)}^{2}+{\left(b*\right)}^{2}}$$

(4) $$\mathrm{Hue}\;\text{angle}=\tan^{-1}\left(\frac{b*}{a*}\right)$$

### 2.8 Phytochemical analysis

#### 2.8.1 Total polyphenol content (TPC)

The total polyphenols of the samples were evaluated using the method of (Akpro et al., 2019) with few modifications. 0.1 mL of the samples were taken in the test tubes, followed by the addition of 6 mL distilled water. Then, 0.5 mL of Folin reagent was added in each test tube separately. Afterwards, 20 % sodium carbonate solution was prepared and 1.5 mL of the prepared solution was added in the test tubes followed by adding 1.9 mL of distilled water. A blank was also prepared in the similar fashion by replacing the sample with distilled water. The test tubes were then incubated at 40 °C for 30 min. The absorbance of the reaction mixtures was then measured using a spectrophotometer at 765 nm. A standard curve of gallic acid was also prepared to evaluate the results. For this purpose, the absorbance of various concentrations (0, 2, 5, 10, 15, 20 mg/100 mL) of gallic acid was taken to plot the graph (concentration versus absorbance) and get the standard curve. Hence, the results were expressed as mg GAE/100 mL of the samples.

#### 2.8.2 Total flavonoid content (TFC)

Total flavonoid content of the samples were evaluated using the methodology of (Kannaian et al., 2020) with few modifications. For this purpose, 5 mL of each sample was taken in test tubes. 2 % aluminium trichloride (made in ethanol) solution was prepared and 5 mL of this solution was then added in the test tubes. A blank was prepared in the similar fashion by replacing the sample with distilled water. The test tubes were incubated at room temperature for 10 min. The absorbance was then measured using a spectrophotometer at 415 nm. A standard curve of quercetin was prepared to evaluate the results. For this, the absorbance of various concentrations (0, 2, 5, 10, 15, 20 mg/100 mL) of quercetin was taken to plot a graph (concentration versus absorbance) and get the standard curve. Hence, the results were expressed as mg QE/100 mL of the samples.

#### 2.8.3 DPPH

DPPH (2,2-diphenyl-1-picrylhydrazyl) radical scavenging effect (Kalina & Navaratne, 2019) was estimated to determine the antioxidant activity of the squash samples. For this, 1 mL of the sample was mixed with 3 mL of 95 % methanol in a test tube followed by the addition of 1 mL of DPPH solution (1 mM; freshly prepared). For the blank preparation, 1 mL of 95 % methanol was added in place of the sample. This reaction mixture served as the control. All the test tubes were vortexed for a minute to mix the solutions properly. The test tubes were then incubated at room temperature for about 10 min. The absorbance of the samples was observed at 517 nm in a spectrophotometer. Total antioxidant activity was quantified by using the following formula:

(5) $$\text{Antioxidant activity}\;\left(\%\right)=\left(\frac{\;A_{\text{control}}-A_{\text{sample}}}{A_{\text{control}}}\right)\times 100$$

where,

A_control_ = Absorbance of the control.

A_sample =_ Absorbance of the sample.

#### 2.8.4 Frap

The FRAP (ferric reducing antioxidant power) assay was performed to estimate the antioxidant capacity of the samples (Phonphoem et al., 2022). To begin with, stock solutions were prepared to make the FRAP reagent. For this, 300 mM acetate and glacial acetic acid buffer (pH = 3.6) was mixed with 20 mM ferric chloride and 10 mM TPTZ (4.6-tripryridylstriazine) (made in 40 mM HCL) solutions. These solutions were mixed in 10:1:1. Afterwards, 300 μl of each sample was taken in separate test tubes and 4.5 mL of the prepared FRAP reagent was added into it. The blank was prepared in the same way by replacing the sample with water. After vortex, test tubes were placed in a water bath at 37 °C for 4 min. The absorbance of the samples was then checked at 593 nm using a spectrophotometer. Ferrous sulfate (10-1000 μM) was used to generate the standard curve and compute the FRAP values of the samples. The results were expressed as μmol Fe^2+^/100 mL of the samples.

### 2.9 Accelerated shelf life assessment of coconut squash

To assess the accelerated shelf life of the prepared product, Q10 methodology was used as outlined by Hemanth et al. (2020). For this purpose, the squash samples were stored at two different temperatures i.e. room temperature (20 °C) and elevated temperature (40 °C). The acid value was chosen as the spoilage factor to predict the shelf life of the squash. The rate of reaction for the acid value was monitored at the specified intervals during the storage at both temperatures. The acid value was calculated using the formula:

(6) $$\mathrm{FFA}\;\text{number}=\frac{\text{Titre value}\times \text{Normality of}\;\mathrm{KOH}\times 200.32}{\text{Weight of sample}\times 1000}\times 100\;$$

(Side et al., 2023)

Accelerated aging time duration (AATD) is the number of days taken by the sample to remain stable at elevated temperature. Using linear regression, the observed shelf life at the elevated conditions was calculated up to value 6 (Food Safety and Standards Authority of India, 2011).

Q10, the factor by which reaction rate increases by 10 °C rise in temperature, was calculated as:

(7) $$Q10=\left(\frac{K2}{K1}\right)\;10/\mathrm{\Delta T}$$

where,

K_1_ = Rate of reaction at room temperature (T1).

K_2_ = Rate of reaction at elevated temperature (T2).

Accelerated aging rate (AAR) was then calculated as:

(8) $$\mathrm{AAR}=Q10^{\mathrm{\Delta T}/10}$$

Using the value of AATD, desired real time (DRT) was estimated as:

(9) $$\mathrm{DRT}=\mathrm{AAR}\times \text{Shelf life}\;\mathrm{at}\;\text{elevated conditions}\;\left(\text{AATD}\right)$$

Thus, by accelerating the spoilage rate of the product by storing it at elevated temperature, the shelf life of the coconut squash at room temperature was predicted.

### 2.10 Sensory evaluation

The sensory evaluation of the squash samples was done by 35 panelists (the students of Kauser Abdulla Malik School of Life Sciences, FCCU, Lahore) using a hedonic rating scale to assess the public acceptance of the drink (Guthale et al., 2023). The panelists were provided with a sensory evaluation form in which the scores were recorded for the sensory attributes i.e. color, taste, aroma, flavor, mouth feel, aftertaste and overall acceptability of the squash samples. According to the hedonic scale, the scores were given as: 9- Like extremely, 8- Like very much, 7- Like moderately, 6- like slightly, 5- Neither like nor dislike, 4- Dislike slightly, 3- Dislike moderately, 2- Dislike very much, 1- Dislike extremely. Five samples of squash, labeled with random codes to avoid any biases, were presented to the panelists for their sensory evaluation. Water was provided to the participants to neutralize the taste in between testing of samples.

### 2.11 Statistical analysis

The obtained data were analyzed statistically using statistix (8.1) software. Analysis of variance (ANOVA) was performed to identify the degree of significance between the treatments. Tukey's honest significance difference test (HSD) was further employed to compare the means. The Kruskal-Wallis test was employed for the evaluation of the sensory results.

## 3 Results

### 3.1 Physicochemical parameters of coconut squash

#### 3.1.1 pH

Statistical analysis revealed highly significant (*p* < 0.01) effect of treatments and storage on the pH of squash at room temperature. The interactions between treatments and storage also behaved highly significant (*p* < 0.01). The mean values for pH at room temperature (20 °C) are shown in Table 2. A decreasing trend in the mean values was observed throughout the storage intervals. The maximum mean value was recorded as 2.83 ± 0.01 at the initial day of the storage followed by 2.82 ± 0.01, 2.76 ± 0.01 and 2.74 ± 0.01 at the 14th, 28th and 42nd day of the storage, respectively. The minimum mean value was recorded as 2.68 ± 0.01 at the 56th day of the storage period. Amongst the treatments, the mean values increased with the increasing concentration of coconut milk from T1 to T5 recorded as 2.51 ± 0.01, 2.66 ± 0.01, 2.79 ± 0.01, 2.87 ± 0.01 and 2.99 ± 0.01 for T1, T2, T3, T4 and T5, respectively. A prominent decrease in pH values was seen in T1 ranging from 2.65 ± 0.01 to 2.36 ± 0.01 at the 1st and 56th day of the storage followed by T4 from 2.92 ± 0.01 to 2.78 ± 0.01, T5 from 3.05 ± 0.01 to 2.92 ± 0.02 and T2 from 2.71 ± 0.01 to 2.61 ± 0.02 at the same respective days. T3 showed the minimum decrease in pH during storage with values ranging from 2.83 ± 0.01 to 2.75 ± 0.01 at the start and end, respectively.

**Table 2:** Means for physicochemical parameters of coconut squash at room temperature (20 °C).

Parameter	Treatments	Storage Intervals (in days)					
		Initial	14th	28th	42nd	56th	Pooled Means
pH	T1	2.65 ± 0.01^lm^	2.64 ± 0.01^lm^	2.47 ± 0.02^o^	2.44 ± 0.01^p^	2.36 ± 0.01^q^	2.51 ± 0.01^e^
	T2	2.71 ± 0.01^j^	2.69 ± 0.01^jk^	2.67 ± 0.01^kl^	2.64 ± 0.01^m^	2.61 ± 0.02^n^	2.66 ± 0.01^d^
	T3	2.83 ± 0.01^f^	2.81 ± 0.01^fg^	2.79 ± 0.01^gh^	2.77 ± 0.01^hi^	2.75 ± 0.01^i^	2.79 ± 0.01^c^
	T4	2.92 ± 0.01^c^	2.91 ± 0.01^cd^	2.89 ± 0.01^de^	2.86 ± 0.01^e^	2.78 ± 0.01^hi^	2.87 ± 0.01^b^
	T5	3.05 ± 0.01^b^	3.03 ± 0.01^c^	2.99 ± 0.01^a^	2.97 ± 0.01^a^	2.92 ± 0.02^b^	2.99 ± 0.01^a^
	Pooled Means	2.83 ± 0.01^a^	2.82 ± 0.01^b^	2.76 ± 0.01^c^	2.74 ± 0.01^d^	2.68 ± 0.01^e^	
Acidity (%)	T1	0.97 ± 0.01^de^	0.99 ± 0.01^d^	1.03 ± 0.01^bc^	1.03 ± 0.01^bc^	1.06 ± 0.01^a^	1.02 ± 0.01^a^
	T2	0.96 ± 0.01^ef^	0.99 ± 0.01^d^	1.02 ± 0.01^c^	1.03 ± 0.01^bc^	1.05 ± 0.01^ab^	1.01 ± 0.01^a^
	T3	0.94 ± 0.01^f^	0.95 ± 0.01^f^	0.98 ± 0.01^de^	0.99 ± 0.01^d^	1.03 ± 0.01^bc^	0.98 ± 0.01^c^
	T4	0.95 ± 0.01^f^	0.96 ± 0.01^ef^	1.01 ± 0.01^c^	1.02 ± 0.01^c^	1.02 ± 0.02^c^	0.99 ± 0.01^b^
	T5	0.94 ± 0.01^f^	0.98 ± 0.01^de^	1.01 ± 0.01^c^	1.02 ± 0.01^c^	1.02 ± 0.01^c^	0.99 ± 0.01^b^
	Pooled Means	0.95 ± 0.01^d^	0.97 ± 0.01^c^	1.01 ± 0.01^b^	1.02 ± 0.01^b^	1.04 ± 0.01^a^	
TSS (°B)	T1	60.52 ± 0.01^n^	61.14 ± 0.02^l^	61.56 ± 0.01^j^	61.09 ± 0.02^m^	62.01 ± 0.01^i^	61.26 ± 0.01^e^
	T2	62.03 ± 0.02^hi^	62.25 ± 0.02^e^	61.51 ± 0.01^k^	62.07 ± 0.01^h^	62.52 ± 0.01^c^	62.08 ± 0.01^d^
	T3	62.01 ± 0.01^i^	62.07 ± 0.02^h^	62.06 ± 0.02^h^	62.21 ± 0.02^f^	62.55 ± 0.01^bc^	62.18 ± 0.02^c^
	T4	61.54 ± 0.01^jk^	62.04 ± 0.02^hi^	62.68 ± 0.01^a^	62.57 ± 0.02^b^	62.56 ± 0.01^bc^	62.28 ± 0.01^b^
	T5	62.12 ± 0.01^g^	62.35 ± 0.02^d^	62.52 ± 0.01^c^	62.53 ± 0.02^c^	62.59 ± 0.01^b^	62.42 ± 0.01^a^
	Pooled Means	61.64 ± 0.01^e^	61.97 ± 0.02^d^	62.07 ± 0.01^c^	62.09 ± 0.02^b^	62.45 ± 0.01^a^	

The mean value with different alphabetical letters are statistically different (*p* < 0.05).

#### 3.1.2 Acidity

Statistical analysis depicted highly significant (*p* < 0.01) effect of treatments, storage and their interactions at room temperature. Table 2 displays the mean values for acidity during storage at room temperature (20 °C). An increasing trend in the mean values was observed throughout the storage intervals. The minimum mean value was recorded as 0.95 ± 0.01 % at the start of the trial followed by 0.97 ± 0.01, 1.01 ± 0.01 and 1.02 ± 0.01 % at the 14th, 28th and 42nd day of the trial, respectively. The maximum mean value was recorded as 1.04 ± 0.01 % at the 56th day of the trial. Amongst the treatments, a decreasing trend in the mean values for acidity was observed. The highest mean value was noted in T1 as 1.02 ± 0.01 % followed by T2 as 1.01 ± 0.01 %. T3 exhibited the lowest mean value recorded as 0.98 ± 0.01 %. T4 and T5 showed the same mean value for acidity as 0.99 ± 0.01 %. The highest rise in acidity was observed in T1 from 0.97 ± 0.01 to 1.06 ± 0.01 %, T2 from 0.96 ± 0.01 to 1.05 ± 0.01 % and T3 from 0.94 ± 0.01 to 1.03 ± 0.01 % from start to the end of the trial, respectively. T4 showed the lowest rise in acidity from 0.95 ± 0.01 % at the initial day to 1.02 ± 0.02 % at the 56th day of the storage followed by T5 from 0.94 ± 0.01 to 1.02 ± 0.01 % at the same respective days.

#### 3.1.3 Total soluble solids (TSS)

Statistical analysis depicted highly significant (*p* < 0.01) effect of treatments, storage and their interactions at room temperature. Table 2 exhibits the mean values for TSS (°B) during the storage at room temperature (20 °C). A gradual increase was visible in the mean values throughout the storage intervals. The minimum mean value was noted as 61.64 ± 0.01°B at the 1st day of storage while the maximum mean value was noted as 62.45 ± 0.01°B at the 56th day of storage. The mean values at the 14th, 28th and 42nd day of the storage were documented as 61.97 ± 0.02, 62.07 ± 0.01 and 62.09 ± 0.02°B, respectively. The treatments also demonstrated a gradual increasing trend in TSS values as indicated by their means. The lowest mean value was recorded as 61.26 ± 0.01°B for T1 whereas the highest mean value was recorded as 62.42 ± 0.01°B for T5. The mean values for T2, T3 and T4 showed nearly the same values recorded as 62.08 ± 0.01, 62.18 ± 0.02 and 62.28 ± 0.01°B, respectively. The highest change in TSS values was noted in T1 from 60.52 ± 0.01°B at the 1st day to 62.01 ± 0.01°B at the 56th day of storage, while T5 showed the lowest change in TSS values ranging from 62.12 ± 0.01 to 62.59 ± 0.01°B at the same respective days of the storage.

### 3.2 Sugar profile of coconut squash

#### 3.2.1 Reducing sugars

Statistical analysis depicted highly significant (*p* < 0.01) effect of treatments, storage and their interactions at room temperature. Table 3 presents the mean values for reducing sugars (mg/100 mL) when the treatments were stored at the room temperature (20 °C). The mean values depicted that the concentration of reducing sugars continuously declined throughout the storage intervals. The maximum mean value was recorded as 177.67 ± 0.82 mg/100 mL at the 1st day of the trial followed by 174.19 ± 0.98, 167.06 ± 1.94 and 148.67 ± 1.08 mg/100 mL at the 14th, 28th and 42nd day of the research trial, respectively. The minimum mean value was recorded as 143.41 ± 0.89 mg/100 mL at the 56th day of the research trial. Amongst the treatments, the concentration of reducing sugars increased with the increasing concentration of coconut milk from T1 to T5. The lowest mean value for reducing sugars was documented in T1 as 157.36 ± 1.16 mg/100 mL followed by T2, T3 and T4 with the mean values of 159.70 ± 1.58, 162.91 ± 0.74 and 164.94 ± 1.23 mg/100 mL, respectively. However, the highest reducing sugars were documented in T5 having the mean value of 166.10 ± 1.00 mg/100 mL. Amongst the treatments, T5 showed the maximum decline in reducing sugars from 184.39 ± 1.13 mg/100 mL at the initial day to 144.54 ± 0.79 mg/100 mL at the 56th day, whereas T2 had the minimum decline from 171.72 ± 0.45 to 143.79 ± 1.01 mg/100 mL at the same respective days.

**Table 3:** Means for sugar profile of coconut squash at room temperature (20 °C).

Parameter	Treatments	Storage Intervals (in days)					
		Initial	14th	28th	42nd	56th	Pooled Means
Reducing Sugars	T1	170.66 ± 0.30^cd^	168.63 ± 0.35^de^	158.24 ± 2.36^f^	147.17 ± 1.00^gh^	142.09 ± 1.81^i^	157.36 ± 1.16^d^
	T2	171.72 ± 0.45^cd^	170.23 ± 0.60^c-e^	166.04 ± 3.56^e^	146.72 ± 2.29^gh^	143.79 ± 1.01^hi^	159.70 ± 1.58^c^
	T3	178.32 ± 1.34^b^	173.14 ± 0.67^c^	170.26 ± 0.63^c-e^	149.81 ± 0.80^g^	143.01 ± 0.24^hi^	162.91 ± 0.74^b^
	T4	183.24 ± 0.89^a^	177.79 ± 1.57^b^	170.20 ± 2.34^c-e^	149.87 ± 0.75^g^	143.62 ± 0.61^hi^	164.94 ± 1.23^a^
	T5	184.39 ± 1.13^a^	181.18 ± 1.72^ab^	170.58 ± 0.81^cd^	149.80 ± 0.57^g^	144.54 ± 0.79^hi^	166.10 ± 1.00^a^
	Pooled Means	177.67 ± 0.82^a^	174.19 ± 0.98^b^	167.06 ± 1.94^c^	148.67 ± 1.08^d^	143.41 ± 0.89^e^	
Non-reducing Sugars (Sucrose)	T1	70.39 ± 0.09^cd^	69.46 ± 0.15^d-f^	65.19 ± 0.54^i^	56.52 ± 0.34^j-l^	54.34 ± 0.45^m^	63.18 ± 0.31^d^
	T2	70.84 ± 0.21^cd^	70.18 ± 0.25^c-e^	65.64 ± 0.73^hi^	56.73 ± 0.55^j-l^	55.17 ± 0.84^lm^	63.71 ± 0.52^d^
	T3	72.73 ± 0.58^b^	71.48 ± 0.29^bc^	67.31 ± 0.78^gf^	57.38 ± 0.51^jk^	55.84 ± 0.87^k-m^	64.95 ± 0.61^c^
	T4	75.95 ± 0.39^a^	72.86 ± 0.93^b^	67.82 ± 0.51^fg^	57.07 ± 0.55^jk^	56.67 ± 0.27^j-l^	66.07 ± 0.53^b^
	T5	75.56 ± 0.79^a^	75.28 ± 0.82^a^	68.51 ± 0.23^e-g^	57.99 ± 0.52^j^	57.25 ± 0.34^jk^	66.92 ± 0.54^a^
	Pooled Means	73.09 ± 0.41^a^	71.85 ± 0.49^b^	66.89 ± 0.56^c^	57.14 ± 0.49^d^	55.85 ± 0.55^e^	
Total Sugars	T1	241.05 ± 0.39^c-e^	238.08 ± 0.50^de^	223.43 ± 2.88^g^	203.69 ± 0.66^hi^	196.43 ± 1.53^k^	220.54 ± 1.19^e^
	T2	242.57 ± 0.66^cd^	240.41 ± 0.85^c-e^	231.68 ± 2.86^f^	203.45 ± 2.81^h-j^	198.96 ± 0.88^jk^	223.41 ± 1.61^d^
	T3	251.05 ± 1.22^b^	244.61 ± 0.95^c^	237.57 ± 1.36^e^	207.18 ± 0.81^h^	198.85 ± 0.77^jk^	227.85 ± 1.02^c^
	T4	259.19 ± 1.28^a^	250.66 ± 1.84^b^	238.01 ± 1.86^de^	206.94 ± 1.29^h^	200.29 ± 0.68^i-k^	231.02 ± 1.39^b^
	T5	259.95 ± 0.50^a^	256.46 ± 2.43^a^	239.10 ± 0.97^de^	207.79 ± 1.04^h^	201.79 ± 1.11^ij^	233.02 ± 1.21^a^
	Pooled Means	250.76 ± 0.81^a^	246.04 ± 1.31^b^	233.96 ± 1.99^c^	205.81 ± 1.32^d^	199.26 ± 0.99^e^	

The mean value with different alphabetical letters are statistically different (*p* < 0.05).

#### 3.2.2 Non-reducing sugars (sucrose)

Statistical analysis depicted highly significant (*p* < 0.01) effect of treatments, storage and their interactions at room temperature. Table 3 presents the mean values of sucrose (mg/100 mL) when the treatments were stored at the room temperature. The mean values depicted that the concentration of sucrose continuously declined throughout the storage intervals. The maximum mean value for sucrose was recorded as 73.09 ± 0.41 mg/100 mL at the 1st day of the trial followed by 71.85 ± 0.49, 66.89 ± 0.56 and 57.14 ± 0.49 mg/100 mL at the 14th, 28th and 42nd day of the research trial, respectively. The minimum mean value was recorded as 55.85 ± 0.55 mg/100 mL at the 56th day of the research trial. Amongst the treatments, the sucrose concentration increased with the increasing concentration of coconut milk from T1 to T5. The lowest sucrose concentration was documented in T1 with the mean value of 63.18 ± 0.31 mg/100 mL followed by T2, T3 and T4 with the mean values of 63.71 ± 0.52, 64.95 ± 0.61 and 66.07 ± 0.53 mg/100 mL, respectively. However, the highest sucrose was documented in T5 having the mean value of 66.92 ± 0.54 mg/100 mL. Amongst the treatments, T4 showed the maximum decline in sucrose concentration from 75.95 ± 0.39 mg/100 mL at the initial day to 56.67 ± 0.27 mg/100 mL at the 56th day, whereas T2 had the minimum decline in sucrose from 70.84 ± 0.21 to 55.17 ± 0.84 mg/100 mL at the same respective days.

#### 3.2.3 Total sugars

Statistical analysis depicted highly significant (*p* < 0.01) effect of treatments, storage and their interactions at room temperature. Table 3 presents the mean values for total sugars (mg/100 mL) when the treatments were stored at the room temperature (20 °C). The mean values depicted that the concentration of total sugars continuously declined throughout the storage intervals. The maximum mean value was recorded as 250.76 ± 0.81 mg/100 mL at the 1st day of the trial followed by 246.04 ± 1.31, 233.96 ± 1.99 and 205.81 ± 1.32 mg/100 mL at the 14th, 28th and 42nd day of the research trial, respectively. The minimum mean value was recorded as 199.26 ± 0.99 mg/100 mL at the 56th day of the research trial. Amongst the treatments, the concentration of total sugars increased with the increasing concentration of coconut milk from T1 to T5. The lowest mean value for total sugars was documented in T1 as 220.54 ± 1.19 mg/100 mL followed by T2, T3 and T4 with the mean values of 223.41 ± 1.61, 227.85 ± 1.02 and 231.02 ± 1.39 mg/100 mL, respectively. However, the highest total sugars were documented in T5 having the mean value of 233.02 ± 1.21 mg/100 mL. Amongst the treatments, T4 showed the maximum decline in total sugars from 259.19 ± 1.28 mg/100 mL at the initial day to 200.29 ± 0.68 mg/100 mL at the 56th day, whereas T2 had the minimum decline from 242.57 ± 0.66 to 198.96 ± 0.88 mg/100 mL at the same respective days.

### 3.3 Color profile of coconut squash

Color profile of the squash is a critical quality attribute that highly influences the general acceptability of the product. The color parameters of coconut squash were assessed through CIELAB color system. The values of L (lightness-darkness), a* (greenish-reddish tint), b* (bluish-yellowish tint), chroma and hue angle were determined to evaluate the color variations during the storage of the treatments. Table 4 displays the mean squares for L, a*, b* chroma and hue angle.

**Table 4:** Means for color profile of coconut squash at room temperature (20 °C).

Parameter	Treatments	Storage Intervals (in days)					
		Initial	14th	28th	42nd	56th	Pooled Means
L	T1	72.03 ± 1.17^a^	67.23 ± 2.06^a-f^	71.57 ± 2.35^a^	70.83 ± 3.78^a-c^	69.17 ± 6.31^a-d^	70.17 ± 3.13^a^
	T2	71.07 ± 0.68^ab^	68.07 ± 0.76^a-e^	68.93 ± 5.55^a-d^	57.03 ± 4.29^e-i^	54.43 ± 3.61^hi^	63.91 ± 2.98^b^
	T3	67.57 ± 0.99^a-f^	58.73 ± 0.35^d-i^	59.77 ± 6.74^c-i^	61.97 ± 4.04^a-i^	61.57 ± 3.41^a-i^	61.92 ± 3.11^bc^
	T4	65.33 ± 1.31^a-h^	61.43 ± 1.65^a-i^	60.13 ± 5.26^b-i^	56.53 ± 4.95^f-i^	55.37 ± 3.54^g-i^	59.76 ± 3.34^c^
	T5	66.13 ± 1.31^a-g^	70.83 ± 1.55^a-c^	59.27 ± 2.14^d-i^	51.83 ± 5.87^i^	53.53 ± 1.53^i^	60.32 ± 2.48^bc^
	Pooled Means	68.43 ± 1.09^a^	65.26 ± 1.27^ab^	63.93 ± 4.41^b^	59.64 ± 4.59^c^	58.81 ± 3.68^c^	
a*	T1	−4.97 ± 0.71	1.33 ± 0.45	−0.47 ± 0.15	0.97 ± 0.12	0.83 ± 0.46	−0.46 ± 0.38^ab^
	T2	−4.53 ± 0.06	0.57 ± 0.15	−1.07 ± 0.75	1.47 ± 1.67	0.27 ± 0.49	−0.66 ± 0.62^b^
	T3	−3.83 ± 0.31	1.17 ± 0.25	−0.47 ± 0.65	2.77 ± 1.45	1.43 ± 1.15	0.21 ± 0.76^a^
	T4	−4.33 ± 0.21	0.83 ± 0.64	−0.93 ± 0.55	1.43 ± 0.21	−0.43 ± 0.76	−0.69 ± 0.47^b^
	T5	−3.77 ± 0.15	0.83 ± 0.35	−0.63 ± 0.45	1.53 ± 0.59	−0.37 ± 0.42	−0.48 ± 0.39^b^
	Pooled Means	−4.29 ± 0.29^d^	0.95 ± 0.37^b^	−0.71 ± 0.51^c^	1.63 ± 0.81^a^	0.35 ± 0.66^b^	
b*	T1	2.23 ± 0.99^f-h^	5.03 ± 0.61^b-e^	3.47 ± 1.04^c-h^	5.07 ± 0.87^b-e^	5.63 ± 2.39^a-e^	4.29 ± 1.18^b^
	T2	2.53 ± 0.32^f-h^	4.03 ± 0.31^c-g^	3.23 ± 1.45^e-h^	7.73 ± 0.51^a^	5.83 ± 0.32^a-d^	4.67 ± 0.58^ab^
	T3	3.77 ± 0.35^c-g^	6.83 ± 0.23^ab^	4.43 ± 0.25^b-f^	6.83 ± 0.35^ab^	4.37 ± 0.92^b-g^	5.25 ± 0.42^a^
	T4	3.83 ± 0.64^c-g^	5.87 ± 0.21^a-c^	4.03 ± 0.31^c-g^	5.63 ± 0.75^a-e^	4.27 ± 0.81^c-g^	4.73 ± 0.54^ab^
	T5	4.17 ± 0.31^c-g^	4.67 ± 0.47^b-f^	3.37 ± 0.42^d-h^	1.13 ± 0.38^h^	1.93 ± 0.59^gh^	3.05 ± 0.43^c^
	Pooled Means	3.31 ± 0.52^c^	5.29 ± 0.37^a^	3.71 ± 0.69^bc^	5.28 ± 0.57^a^	4.41 ± 1.01^b^	
Chroma	T1	5.51 ± 0.61^a-e^	5.21 ± 0.71^b-e^	3.51 ± 1.00^d-g^	5.16 ± 0.84^b-e^	5.73 ± 2.30^a-e^	5.02 ± 1.09^a^
	T2	5.20 ± 0.13^b-e^	4.08 ± 0.29^d-g^	3.41 ± 1.61^e-g^	7.99 ± 0.58^a^	5.85 ± 0.31^a-e^	5.31 ± 0.58^a^
	T3	5.38 ± 0.21^b-e^	6.94 ± 0.19^a-c^	4.49 ± 0.24^c-f^	7.45 ± 0.75^ab^	4.65 ± 1.17^c-e^	5.78 ± 0.51^a^
	T4	5.79 ± 0.52^a-e^	5.95 ± 0.25^a-d^	4.17 ± 0.17^d-g^	5.82 ± 0.74^a-e^	4.34 ± 0.78^d-g^	5.21 ± 0.49^a^
	T5	5.63 ± 0.04^a-e^	4.75 ± 0.53^c-e^	3.45 ± 0.42^d-g^	1.94 ± 0.56^g^	1.99 ± 0.61^fg^	3.55 ± 0.43^b^
	Pooled Means	5.50 ± 0.30^a^	5.39 ± 0.39^a^	3.81 ± 0.69^b^	5.67 ± 0.69^a^	4.51 ± 1.03^b^	
Hue	T1	−0.42 ± 0.20	1.31 ± 0.06	−1.42 ± 0.09	1.38 ± 0.06	1.39 ± 0.16	0.45 ± 0.11
	T2	−0.51 ± 0.06	1.43 ± 0.04	−1.29 ± 0.11	1.39 ± 0.21	0.47 ± 1.73	0.30 ± 0.43
	T3	−0.78 ± 0.07	1.40 ± 0.04	−0.42 ± 1.69	1.20 ± 0.18	1.29 ± 0.21	0.54 ± 0.44
	T4	−0.72 ± 0.07	1.43 ± 0.11	−1.34 ± 0.15	1.32 ± 0.04	−0.42 ± 1.70	0.05 ± 0.41
	T5	−0.83 ± 0.08	1.40 ± 0.06	−1.39 ± 0.13	0.64 ± 0.23	−0.35 ± 1.61	−0.11 ± 0.42
	Pooled Means	−0.65 ± 0.10^c^	1.39 ± 0.06^a^	−1.17 ± 0.43^c^	1.19 ± 0.14^a^	0.48 ± 1.08^b^	

The mean value with different alphabetical letters are statistically different (*p* < 0.05).

#### 3.3.1 L

Statistical analysis depicted highly significant (*p* < 0.01) effect of treatments, storage and their interactions at room temperature. Table 4 displays the mean values for L at the room temperature (20 °C) storage of the treatments. It was inferred that a continuous decline in the L values was observed during the storage intervals. The maximum mean value for L was recorded as 68.43 ± 1.09 at the initial day of the storage. The mean values at the 14th, 28th and 42nd day of the storage were recorded as 65.26 ± 1.27, 63.93 ± 4.41 and 59.64 ± 4.59, respectively. The lowest mean value was recorded as 58.81 ± 3.68 at the 56th day of the storage. The treatments showed an increasing trend in the mean values for L. The highest mean value for L value was documented in T1 as 70.17 ± 3.13 followed by T2 and T3 with the mean values of 63.91 ± 2.98 and 61.92 ± 3.11, respectively. The lowest mean value for L was observed to be 59.76 ± 3.34 for T4 followed by 60.32 ± 2.48 for T5. Thus, it was inferred that T1 was lightest in color while T4 was darkest in color.

### 3.4 A*

Statistical analysis revealed highly significant (*p* < 0.01) effect of treatments and storage, but they had non-significant interactions at room temperature (*p* > 0.05). Table 4 displays the mean values for a* at the room temperature storage of the treatments. The minimum mean value for a* was recorded as −4.29 ± 0.29 at the initial day while the maximum mean value was recorded as 1.63 ± 0.81 at the 42nd day of the research trial. The mean values at the 14th, 28th and 56th day were reported as 0.95 ± 0.37, −0.71 ± 0.51 and 0.35 ± 0.66, respectively. Amongst the treatments, the highest a* value was documented in T3 with the mean value of 0.21 ± 0.76, while the lowest mean value was noted in T4 as −0.69 ± 0.47. The mean values for T1, T2 and T5 were noted as −0.46 ± 0.38, −0.66 ± 0.62 and − 0.48 ± 0.39, respectively.

### 3.5 B*

Statistical analysis revealed highly significant (*p* < 0.01) effect of treatments, storage and their interactions. Table 4 displays the mean values for b* at the room temperature storage of the treatments. The minimum mean value for b* was recorded as 3.31 ± 0.52 at the initial day of the storage while the maximum mean value was recorded as 5.29 ± 0.37 at the 14th day of the storage. The mean values at the 14th, 28th and 56th day were reported as 3.71 ± 0.69, 5.28 ± 0.57 and 4.41 ± 1.01, respectively. Amongst the treatments, the highest b* value was documented in T3 with the mean value of 5.25 ± 0.42, while the lowest mean value was noted in T5 as 3.05 ± 0.43. The mean values for T1, T2 and T4 were noted as 4.29 ± 1.18, 4.67 ± 0.58 and 4.73 ± 0.54, respectively.

#### 3.5.1 Chroma

Statistical analysis revealed highly significant (*p* < 0.01) effect of treatments, storage and their interactions. Table 4 displays the mean values for chroma at the room temperature storage of the treatments. The minimum mean value for chroma was recorded as 3.81 ± 0.69 at the 28th day of the storage while the maximum mean value was recorded as 5.67 ± 0.69 at the 42nd day of the storage, while the. The mean values at the initial, 14th and 56th day were reported as 5.50 ± 0.30, 5.39 ± 0.39 and 4.51 ± 1.03 respectively. Amongst the treatments, the highest chroma was documented in T3 with the mean value of 5.78 ± 0.51, while the lowest mean value was noted in T5 as 3.55 ± 0.43. T1, T2 and T4 showed the mean values of 5.02 ± 1.09, 5.31 ± 0.58 and 5.21 ± 0.49, respectively.

#### 3.5.2 Hue

At room temperature, statistical analysis revealed highly significant (*p* < 0.01) effect of storage, while treatments behaved non-significantly (*p* > 0.05). Also, the treatments and storage had non-significant (*p* > 0.05) interactions at room temperature. Table 4 displays the mean values for hue angle at the room temperature storage of the treatments. The maximum mean value for hue angle was recorded as 1.39 ± 0.06 at the 14th day of the storage, while the minimum mean value was recorded as −1.17 ± 0.43 at the 28th day of the storage. The mean values at the initial, 42nd and 56th day were reported as −0.65 ± 0.10, 1.19 ± 0.14, 0.48 ± 1.08, respectively. Amongst the treatments, the highest hue angle was documented in T3 with the mean value of 0.54 ± 0.44, while the lowest mean value was noted in T5 as −0.11 ± 0.42. The mean values for T1, T2 and T4 were noted as 0.45 ± 0.11, 0.30 ± 0.43 and 0.05 ± 0.41, respectively.

### 3.6 Phytochemical parameters of coconut squash

#### 3.6.1 Total polyphenol content (TPC)

Statistical analysis depicted highly significant (*p* < 0.01) effect of treatments, storage and their interactions at room temperature. The mean values of TPC (mg GAE/100 mL) are shown in Table 5 when the treatments were stored at the room temperature. It was inferred that a continuous decline in the mean values was observed throughout the storage intervals of the research trial. The maximum mean value for the polyphenol content was recorded as 785.59 ± 1.72 mg GAE/100 mL at the 1st day followed by 694.93 ± 1.93, 491.49 ± 2.71 and 231.28 ± 1.48 mg GAE/100 mL at the 14th, 28th and 42nd day of the research trial, respectively. The minimum mean value was recorded as 211.98 ± 1.33 mg GAE/100 mL at the 56th day of the research trial. The treatments showed an increasing trend in polyphenol content with the increasing concentration of coconut milk from T1 to T5. The lowest polyphenol content was documented in T1 as shown by the mean value of 297.23 ± 1.66 mg GAE/100 mL followed by T2, T3 and T4 as 437.17 ± 2.31, 515.72 ± 1.71 and 569.46 ± 1.64 mg GAE/100 mL, respectively. The highest polyphenol content was observed to be 595.68 ± 1.86 mg GAE/100 mL for T5. Amongst the treatments, T2 showed the maximum decline in polyphenol content from 805.09 ± 2.84 mg GAE/100 mL at the initial day to 176.61 ± 1.16 mg GAE/100 mL at the 56th day, whereas T1 had the minimum decline in polyphenol content from 586.95 ± 1.29 to 103.17 ± 1.81 mg GAE/100 mL at the same respective days.

**Table 5:** Means for phytochemical parameters of coconut squash at room temperature (20 °C).

Parameter	Treatments	Storage Intervals (in days)					
		Initial	14th	28th	42nd	56th	Pooled Means
TPC	T1	586.95 ± 1.29^h^	375.63 ± 0.65^j^	296.11 ± 3.37^l^	124.27 ± 1.18^r^	103.17 ± 1.81^s^	297.23 ± 1.66^e^
	T2	805.09 ± 2.84^d^	643.85 ± 2.41^f^	377.75 ± 1.48^j^	182.57 ± 3.66^q^	176.61 ± 1.16^q^	437.17 ± 2.31^d^
	T3	826.99 ± 0.64^c^	793.53 ± 2.37^e^	507.91 ± 3.57^i^	234.91 ± 0.33^o^	215.27 ± 1.62^p^	515.72 ± 1.71^c^
	T4	843.38 ± 1.81^b^	821.73 ± 1.72^c^	628.63 ± 2.31^g^	285.62 ± 1.29^m^	267.96 ± 1.07^n^	569.46 ± 1.64^b^
	T5	865.53 ± 2.02^a^	839.92 ± 2.52^b^	647.05 ± 2.83^f^	329.03 ± 0.93^k^	296.89 ± 0.98^l^	595.68 ± 1.86^a^
	Pooled Means	785.59 ± 1.72^a^	694.93 ± 1.93^b^	491.49 ± 2.71^c^	231.28 ± 1.48^d^	211.98 ± 1.33^e^	
TFC	T1	72.33 ± 0.19^v^	75.54 ± 0.19^u^	101.81 ± 0.77^o^	112.14 ± 0.89^n^	248.81 ± 0.93^h^	122.13 ± 0.59^e^
	T2	78.38 ± 0.09^tu^	82.78 ± 0.18^s^	115.62 ± 0.56^m^	245.05 ± 0.96^i^	300.05 ± 1.11^f^	164.38 ± 0.58^d^
	T3	79.14 ± 0.15^t^	84.71 ± 0.23^s^	128.16 ± 2.61^l^	296.11 ± 0.86^g^	357.18 ± 0.96^e^	189.06 ± 0.96^c^
	T4	89.34 ± 0.22^r^	94.67 ± 0.21^q^	140.23 ± 0.65^k^	360.17 ± 1.72^d^	379.85 ± 0.67^b^	212.85 ± 0.69^b^
	T5	97.89 ± 0.47^p^	99.16 ± 0.79^op^	235.55 ± 1.09^j^	367.21 ± 0.79^c^	385.97 ± 0.75^a^	237.16 ± 0.78^a^
	Pooled Means	83.42 ± 0.22^e^	87.37 ± 0.32^d^	144.27 ± 1.14^c^	276.14 ± 1.04^b^	334.37 ± 0.88^a^	
DPPH	T1	77.99 ± 0.75^i-l^	76.66 ± 0.31^l-n^	75.32 ± 0.36^no^	69.38 ± 0.35^p^	67.93 ± 0.56^q^	73.46 ± 0.47^e^
	T2	79.11 ± 0.83^hi^	78.27 ± 0.12^i-k^	77.29 ± 0.33^j-l^	75.63 ± 0.08^m-o^	74.23 ± 0.17^o^	76.91 ± 0.31^d^
	T3	86.53 ± 0.28^d^	85.39 ± 0.18^de^	83.83 ± 0.45^f^	78.43 ± 0.47^ij^	76.91 ± 0.24^k-m^	82.22 ± 0.32^c^
	T4	93.97 ± 0.18^b^	92.95 ± 0.81^bc^	91.63 ± 0.44^c^	81.15 ± 0.46^g^	79.91 ± 0.26^gh^	87.92 ± 0.43^b^
	T5	96.86 ± 0.67^a^	96.54 ± 0.68^a^	95.99 ± 0.23^a^	84.71 ± 0.42^ef^	84.19 ± 0.33^ef^	91.66 ± 0.47^a^
	Pooled Means	86.89 ± 0.54^a^	85.96 ± 0.42^b^	84.81 ± 0.36^c^	77.86 ± 0.36^d^	76.63 ± 0.31^e^	
FRAP	T1	1155.28 ± 2.29^j^	1013.17 ± 1.72^n^	755.88 ± 3.52^q^	502.25 ± 2.65^u^	404.58 ± 3.68^w^	766.23 ± 2.77^e^
	T2	1271.71 ± 0.98^h^	1155.29 ± 2.28^j^	928.22 ± 2.65^p^	697.35 ± 0.96^s^	487.32 ± 2.86^v^	907.98 ± 1.95^d^
	T3	1342.63 ± 2.23^g^	1244.41 ± 3.06^i^	1060.78 ± 1.29^m^	732.39 ± 2.22^r^	545.05 ± 2.74^t^	985.05 ± 2.31^c^
	T4	1952.18 ± 1.55^b^	1878.72 ± 1.86^c^	1513.37 ± 2.06^e^	1126.71 ± 2.76^k^	975.72 ± 2.72^o^	1489.34 ± 2.19^b^
	T5	1995.94 ± 2.55^a^	1954.82 ± 2.74^b^	1631.89 ± 1.81^d^	1485.59 ± 2.85^f^	1091.69 ± 1.97^l^	1631.99 ± 2.38^a^
	Pooled Means	1543.55 ± 1.92^a^	1449.28 ± 2.33^b^	1178.03 ± 2.27^c^	908.86 ± 2.29^d^	700.87 ± 2.79^e^	

The mean value with different alphabetical letters are statistically different (*p* < 0.05).

#### 3.6.2 Total flavonoid content (TFC)

Statistical analysis depicted highly significant (*p* < 0.01) effect of treatments, storage and their interactions at room temperature. Table 5 displays the mean values of TFC (mg QE/100 mL) at the room temperature storage of the treatments. It was inferred that a continuous increase in the mean values of total flavonoid content was observed throughout the storage intervals of the research trial. The minimum mean value for the flavonoid content was recorded as 83.42 ± 0.22 mg QE/100 mL at the 1st day followed by 87.37 ± 0.32, 144.27 ± 1.14 and 276.14 ± 1.04 mg QE/100 mL at the 14th, 28th and 42nd day of the research trial, respectively. The maximum mean value was recorded as 334.37 ± 0.88 mg QE/100 mL at the 56th day of the research trial. For the treatments, a continuous increasing trend in the total flavonoid content was observed from T1 to T5. The highest flavonoid content was documented in T5 as 237.16 ± 0.78 mg QE/100 mL followed by T4 as 212.85 ± 0.69 mg QE/100 mL. The lowest flavonoid content was observed to be 122.13 ± 0.59 mg QE/100 mL for T1 followed by 164.38 ± 0.58 and 189.06 ± 0.96 mg QE/100 mL for T2 and T3, respectively. Amongst the treatments, T4 showed the highest rise in flavonoid content from 89.34 ± 0.22 mg QE/100 mL at the initial day to 379.85 ± 0.67 mg QE/100 mL at the 56th day, whereas T1 had the lowest rise in flavonoid content from 72.33 ± 0.19 to 248.81 ± 0.93 mg QE/100 mL at the same respective days.

#### 3.6.3 DPPH

Statistical analysis depicted highly significant (*p* < 0.01) effect of treatments, storage and their interactions at room temperature. Table 5 displays the mean values of DPPH antioxidant activity (%) at the room temperature storage of the treatments. A decreasing trend in the antioxidant activity was observed during the storage. The maximum mean value was noted at the initiation of the trial as 86.89 ± 0.54 %. Afterwards, the activity decreased up to 85.96 ± 0.42, 84.81 ± 0.36 and 77.86 ± 0.36 % at the 14th, 28th and 42nd day of the trial, respectively. The minimum activity was recorded as 76.63 ± 0.31 % at the 56th day i.e. termination of the trial. The treatments showed an increase in the DPPH antioxidant activity with the increasing concentration of coconut milk from T1 to T5 as shown by the mean values. The highest antioxidant activity was documented in T5 as 91.66 ± 0.47 % followed by T4 as 87.92 ± 0.43 %. However, the lowest antioxidant activity was observed to be 73.46 ± 0.47 % for T1 followed by 76.91 ± 0.31 and 82.22 ± 0.32 % for T2 and T3, respectively. Amongst the treatments, a prominent decrease in the antioxidant activity was documented in T4 from 93.97 ± 0.18 % at the initiation to 79.91 ± 0.26 % at the 56th day, whereas the minimum change in antioxidant activity was noted in T2 from 79.11 ± 0.83 to 74.23 ± 0.17 % at the same respective days.

#### 3.6.4 Frap

Statistical analysis depicted highly significant (*p* < 0.01) effect of treatments, storage and their interactions at room temperature. The mean values for FRAP assay (μmol Fe^2+^/100 mL) are shown in Table 5 when stored at room temperature. A decreasing trend in the FRAP values was observed during the storage. The maximum mean value was noted at the initiation of the trial as 1543.55 ± 1.92 μmol Fe^2+^/100 mL. Afterwards, the values decreased up to 1449.28 ± 2.33, 1178.03 ± 2.27 and 908.86 ± 2.29 μmol Fe^2+^/100 mL at the 14th, 28th and 42nd day of the trial, respectively. The minimum value was recorded as 700.87 ± 2.79 μmol Fe^2+^/100 mL at the 56th day i.e. termination of the trial. The treatments showed an increase in the FRAP values with the increasing concentration of coconut milk from T1 to T5 as shown by the mean values. The highest mean value was documented in T5 as 1631.99 ± 2.38 μmol Fe^2+^/100 mL followed by T4 as 1489.34 ± 2.19 μmol Fe^2+^/100 mL. However, the lowest value was observed to be 766.23 ± 2.77 μmol Fe^2+^/100 mL for T1 followed by 907.98 ± 1.95 and 985.05 ± 2.31 μmol Fe^2+^/100 mL for T2 and T3, respectively. Amongst the treatments, a prominent decrease in the FRAP values was documented in T4 from 1952.18 ± 1.55 μmol Fe^2+^/100 mL at the initiation to 975.72 ± 2.72 μmol Fe^2+^/100 mL at the 56th day, whereas the minimum change was noted in T1 from 1155.28 ± 2.29 to 404.58 ± 3.68 μmol Fe^2+^/100 mL at the same respective days.

### 3.7 Accelerated shelf life assessment of coconut squash

Accelerated shelf life testing (ASLT) of the food products is conducted by altering the normal storage conditions of the product to accelerate the deterioration rate that normally take place over the product's shelf life. In the present study, ASLT for coconut squash was conducted to predict its shelf life at room temperature (20 °C). For this purpose, temperature was chosen as acceleration factor and acid value as spoilage factor. The squash treatments were stored at an elevated temperature of 40 °C and their acid value was monitored at specified intervals to predict the deterioration rate at the room temperature using the relationship between temperature and deterioration rate.

#### 3.7.1 Free fatty acid value (%)

Statistical analysis depicted highly significant (*p* < 0.01) effect of treatments, storage and their interactions at room temperature and elevated temperature. The mean values for FFA (%) at 20 °C are displayed in Table 6. An increasing trend in the mean values was observed throughout the storage of the treatments. The minimum mean value was recorded as 3.43 ± 0.15 % at the start of the trial followed by 3.73 ± 0.17, 3.91 ± 0.15 and 4.25 ± 0.14 % at the 14th, 28th and 42nd day of the trial, respectively. The maximum mean value was recorded as 4.64 ± 0.20 % at the last day of the trial. The treatments also exhibited an increasing trend in FFA mean values with increasing concentration of coconut milk from T1 to T5. Amongst the treatments, the lowest mean value was recorded as 3.86 ± 0.16 % for T1, while the highest mean value was observed to be 4.12 ± 0.17 % for T5. The mean values for T2, T3 and T4 were recorded as 3.95 ± 0.17, 3.99 ± 0.17 and 4.04 ± 0.14 %, respectively. The highest rise in FFA% was observed in T2 from 3.33 ± 0.15 % at the initial day to 4.73 ± 0.15 % at the 56th day followed by T3 from 3.37 ± 0.15 to 4.67 ± 0.25 % and T1 from 3.23 ± 0.15 to 4.43 ± 0.25 % at the same respective days. The lowest rise in FFA% was documented in T4 from 3.57 ± 0.15 to 4.63 ± 0.21 % and T5 from 3.67 ± 0.15 to 4.73 ± 0.15 % from start to the end of the storage intervals, respectively.

**Table 6:** Means for free fatty acid value of coconut squash.

Room temperature (20 °C)	Treatments	Storage Intervals (in days)					
		Initial	14th	28th	42nd	56th	Pooled Means
	T1	3.23 ± 0.15	3.67 ± 0.15	3.83 ± 0.06	4.13 ± 0.21	4.43 ± 0.25	3.86 ± 0.16^b^
	T2	3.33 ± 0.15	3.67 ± 0.25	3.73 ± 0.15	4.27 ± 0.15	4.73 ± 0.15	3.95 ± 0.17^ab^
	T3	3.37 ± 0.15	3.77 ± 0.15	3.93 ± 0.15	4.23 ± 0.15	4.67 ± 0.25	3.99 ± 0.17^ab^
	T4	3.57 ± 0.15	3.77 ± 0.15	3.97 ± 0.12	4.27 ± 0.06	4.63 ± 0.21	4.04 ± 0.14^a^
	T5	3.67 ± 0.15	3.77 ± 0.15	4.07 ± 0.25	4.37 ± 0.15	4.73 ± 0.15	4.12 ± 0.17^a^
	Pooled Means	3.43 ± 0.15^e^	3.73 ± 0.17^d^	3.91 ± 0.15^c^	4.25 ± 0.14^b^	4.64 ± 0.20^a^	
Elevated temperature (40 °C)	Treatments	Storage Intervals (in days)					
		Initial	14th	28th	42nd	56th	Pooled Means
	T1	3.33 ± 0.15	3.67 ± 0.12	3.77 ± 0.12	4.27 ± 0.06	4.73 ± 0.15	3.95 ± 0.12^c^
	T2	3.53 ± 0.15	3.67 ± 0.21	3.83 ± 0.06	4.33 ± 0.12	4.77 ± 0.15	4.03 ± 0.14^bc^
	T3	3.53 ± 0.12	3.77 ± 0.06	3.83 ± 0.12	4.53 ± 0.15	4.67 ± 0.15	4.07 ± 0.12^bc^
	T4	3.53 ± 0.15	3.83 ± 0.12	4.17 ± 0.25	4.53 ± 0.15	4.77 ± 0.12	4.17 ± 0.16^ab^
	T5	3.67 ± 0.06	3.87 ± 0.06	4.33 ± 0.15	4.47 ± 0.12	4.77 ± 0.15	4.22 ± 0.11^a^
	Pooled Means	3.52 ± 0.13^e^	3.76 ± 0.11^d^	3.99 ± 0.14^c^	4.43 ± 0.12^b^	4.74 ± 0.14^a^	

The mean value with different alphabetical letters are statistically different (*p* < 0.05).

The mean values for FFA% at 40 °C are displayed in Table 6. An increasing trend in the mean values was observed throughout the storage. The minimum mean value was recorded as 3.52 ± 0.13 % at the start of the trial followed by 3.76 ± 0.11, 3.99 ± 0.14 and 4.43 ± 0.12 % at the 14th, 28th and 42nd day of the trial, respectively. The maximum mean value was recorded as 4.74 ± 0.14 % at the last day of the trial. The treatments also exhibited an increasing trend in FFA mean values with increasing concentration of coconut milk from T1 to T5. Amongst the treatments, the lowest mean value was recorded as 3.95 ± 0.12 % for T1, whereas the highest mean value was observed to be 4.22 ± 0.11 % for T5. The mean values for T2, T3 and T4 were recorded as 4.03 ± 0.14, 4.07 ± 0.12 and 4.17 ± 0.16 %, respectively. The highest rise in FFA% was observed in T1 from 3.33 ± 0.15 to 4.73 ± 0.15 % at the start and end of storage, respectively. T2 and T4 showed nearly the same trend with the values ranging from 3.53 ± 0.15 and 3.53 ± 0.15 % at the initiation to 4.77 ± 0.15 and 4.77 ± 0.12 % at the 56th day, respectively. T5 showed the lowest rise in FFA% from 3.67 ± 0.06 % at the initial day to 4.77 ± 0.15 % at the 56th day of the storage intervals followed by T3 from 3.53 ± 0.12 to 4.67 ± 0.15 % at the same respective intervals.

#### 3.7.2 Expected shelf life of coconut squash

**Fig. 1:**
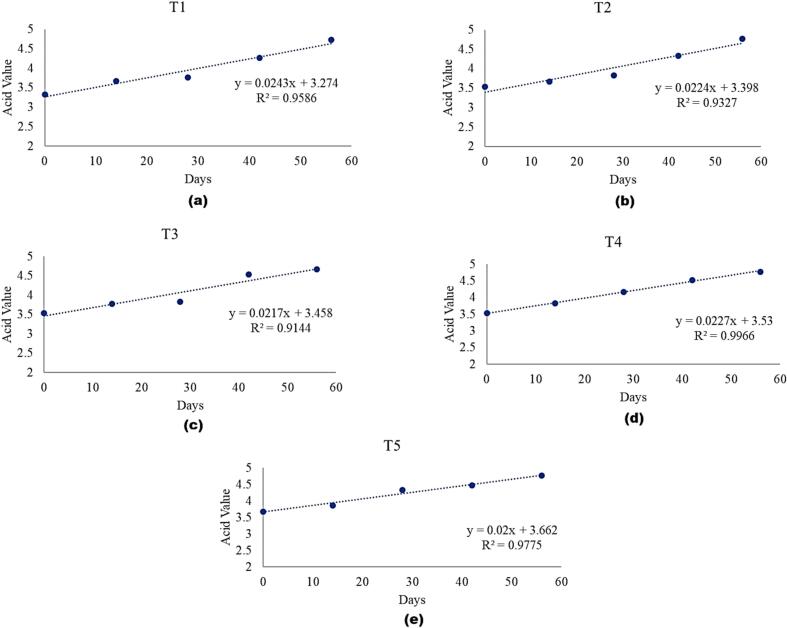
Expected shelf-life with respect to acid value.

The expected shelf life of all the treatments at room temperature are shown in Table 7. An increasing trend in the acid value was observed throughout the storage of treatments at both temperatures. The shelf life of the treatments at elevated temperature was calculated using linear regression (Fig. 1). Amongst the treatments, T1 had the longest expected shelf life of 136 days followed by T4, T5, T3 and T2 having the shelf lives of 127, 121, 103 and 102 days, respectively. These results showed that the T2 had the shortest expected shelf life i.e. 102 days.

**Table 7:** Accelerated shelf life assessment.

Treatments	Initial Acid Value (20 °C)	Final Acid Value (20 °C)	Rate of Reaction (K_1_)	Initial Acid Value (40 °C)	Final Acid Value (40 °C)	Rate of Reaction (K_2_)	Q10	Accelerated Aging Rate (AAR)	Shelf Life at Elevated Conditions (40 °C)	Expected Shelf Life (DRT)
T1	3.23	4.43	1.20	3.33	4.73	1.40	1.08	1.17	116.50	136.31
T2	3.33	4.73	1.40	3.53	4.77	1.24	0.94	0.88	116.16	102.22
T3	3.37	4.67	1.30	3.53	4.67	1.14	0.94	0.88	117.14	103.08
T4	3.57	4.63	1.06	3.53	4.77	1.24	1.08	1.17	108.81	127.31
T5	3.67	4.73	1.06	3.67	4.77	1.10	1.02	1.04	116.90	121.58

### 3.8 Sensory evaluation of coconut squash

Sensory response of food products is important to assess its general acceptance. A food product having an appealing appearance, color, aroma, taste, flavor and texture is preferred for consumption. The coconut squash was subjected to sensory evaluation for color, taste, aroma, texture, aftertaste, flavor, mouth feel and overall acceptability using a 9-point hedonic scale to choose the best treatment based on public preference.

The obtained scores for all the sensory parameters were subjected to statistical analysis. The *p*-value depicted that the distribution of texture, mouth feel, color and flavor was non-significant (*P* > 0.05) across all categories of treatments. However, the distribution of taste, aftertaste and aroma showed significant variations (*P* < 0.05) amongst the treatments. Also, the overall acceptability of all the treatments of coconut squash showed significant (P < 0.05) variations.

**Fig. 2:**
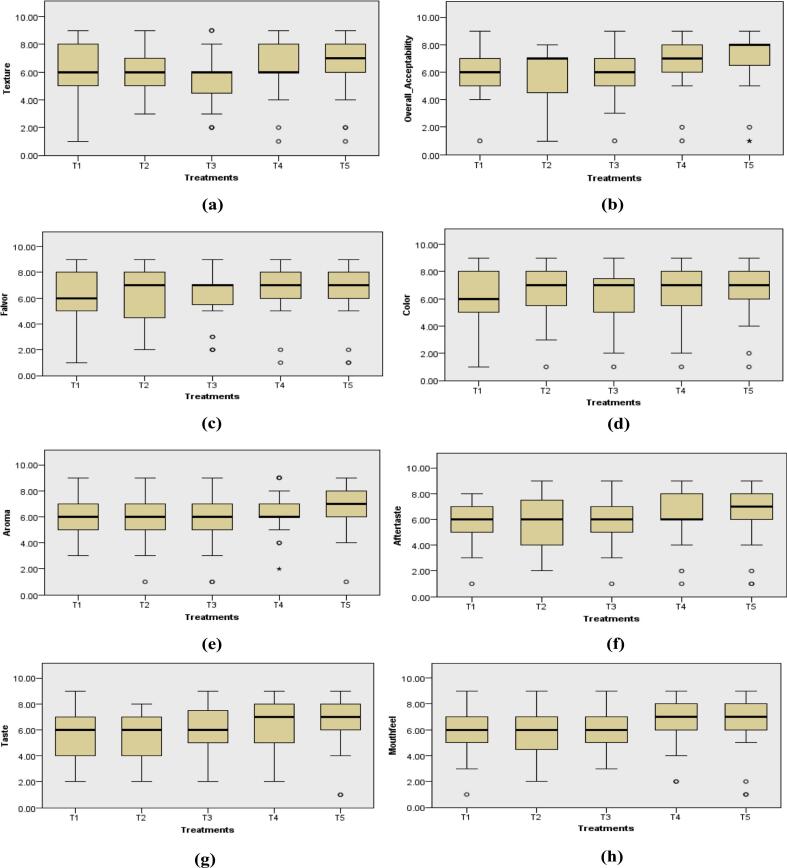
Mean sensory scores for organoleptic properties.

The box and whisker plot for texture is shown in Fig. 2a. From the Fig. 2a, it can be inferred that T5 showed the highest mean rank value as 101.30 followed by T4 as 95.13. The lowest mean rank was shown in T3 as 71.71 followed by T2 and T1 as 82.23 and 89.63, respectively. Thus, T5 had the highest acceptability in terms of the texture of the squash.

Fig. 2b shows the box and whisker plot for the overall acceptability of the squash. The Fig. 2b indicated that T5 had the highest mean rank value as 111.50 followed by T4 as 100.24. The means ranks for T2 and T3 were 75.83 and 78.13, respectively. However, the lowest mean rank was shown in T1 as 74.30. It can be inferred that T5 had the highest overall acceptability amongst all the treatments of the squash.

From the box and whisker plot for the taste profile of coconut squash (Fig. 2g), it can be observed that T5 showed the highest mean rank value as 106.84 followed by T4 as 96.77. The lowest mean rank was shown in T2 as 75.00 followed by T1 and T3 as 75.86 and 85.53, respectively. So, T5 was a preferable choice for consumers in terms of taste of squash.

The highest mean rank for the mouth feel of the squash was shown by T5 having the value of 104.20, as shown by the box and whisker plot in Fig. 2h. The lowest mean rank was shown in T3 as 74.56 followed by T1, T2 and T4 as 78.56, 85.17 and 97.51, respectively. Thus, T5 had the highest acceptability in terms of the mouth feel of the squash.

The scores for aftertaste of squash is shown in the box and whisker plot (Fig. 2f). From the Fig. 2f, it can be inferred that T4 showed the highest mean rank value as 102.33 followed by T5 having nearly the same value as 102.10. The lowest mean rank was shown in T1 as 75.56 followed by T2 and T3 as 77.24 and 82.77, respectively. As a result, T4 and T5 were the most acceptable treatments considering the aftertaste of squash.

The box and whisker plot for aroma of coconut squash is shown in Fig. 2e. From the Fig. 2e, it is clear that T5 showed the highest mean rank value as 113.44 followed by T4 as 93.17. The lowest mean rank was shown in T2 as 73.43 followed by T3 and T1 as 73.63 and 86.33, respectively. Thus, T5 had the highest acceptability in terms of the aroma of the squash.

Fig. 2d shows the box and whisker for color. The Fig. 2d revealed that T4 showed the highest mean rank value as 98.46 followed by T5 as 91.59. The lowest mean rank was documented in T1 as 78.87 followed by T3 and T2 as 81.76 and 89.33, respectively. Hence, for the color profile of squash, T4 got the maximum score.

Fig. 2c displays the mean ranks for the flavor profile of coconut squash treatments. The box and whisker plot showed that T5 got the highest mean rank value as 104.86 followed by T4 as 97.21. The lowest mean rank was noted in T1 as 76.76 followed by T3 and T2 as 78.11 and 83.06, respectively. The maximum score for T5 depicted its highest acceptability for the flavor profile of the coconut squash.

## 4 Discussion

The pH and acidity variations in coconut milk, during storage, are important to monitor, as they directly affect its physicochemical and microbiological stability. In the present study, a decreasing trend in the pH values was observed with increasing acidity when the treatments were stored at room temperature. Similar findings were stated by Kefayatullah et al. (2019) in which the quality evaluation of strawberry squash was conducted They reported a decline in pH and rise in acidity of the strawberry squash treatments when stored at ambient temperature for a period of 3 months. Likewise, Saleem et al. (2020) reported the same trends for pH and titrable acidity in a storage study carried out for the quality evaluation of peach squash. Jayanetti et al. (2023) conducted a study in which industrially pasteurized coconut milk was analyzed for 5 days to monitor the physicochemical changes during this period. As the days progressed, pH values dropped while acidity in the milk increased due to the breakdown of carbohydrates into organic acids. These findings are in accordance to the results of the present study. In another study conducted by Tarek et al. (2020), physicochemical changes in pasteurized coconut milk were monitored during its refrigerated storage for 3 weeks. They reported that pH values declined and the acidity of the samples increased during the storage period due to the production of acetic acid and lactic acid. For the present study, we can conclude that the thermal breakdown of sugars into acids might be the reason for pH decline and the upsurge in acidity of the samples.

The present study has shown an increase in the TSS values during the storage period of the treatments at room temperature. This increase may be possible due to the breakdown of complex polysaccharides into simple sugars as also mentioned by Sethy et al. (2018). They analyzed the changes in the physicochemical properties of commercial fruit beverages during their storage of 9 months at room temperature. Pineapple squash, pineapple syrup and orange squash showed an increase in TSS content during their storage due to the conversion of polysaccharides into monosaccharides and oligosaccharides. Similar results were reported by Din et al. (2019) in which an increase in the TSS content of the mango and guava blended squash was observed during its storage at room temperature. Moreover, the findings of the instant research showed an increasing trend in TSS values with increasing concentrations of coconut milk in the treatments. The rise in TSS is due to the contribution of additional sugars present in coconut milk. These results are in agreement with the findings of Matin et al. (2020) in which the effect of adding coconut milk on the physicochemical properties of yogurt was studied. They mixed different proportions of cow milk and coconut milk to prepare the treatments and stored at 4 °C for the analysis. The results showed an increase in the TSS values with increasing concentration of coconut milk in the treatments.

The outcomes of this study showed a decreasing trend in the total sugar (reducing and non-reducing) content of the squash during its storage at room temperature. Chemical reactions might be the reason for the decline in the sugar content of coconut squash. A study was conducted by Modgil et al. (2019) in which the compositional changes in papaya and mango squash were analyzed during their storage at room temperature for 6 months. They reported a decline in total sugars during storage due to the chemical reactions of sugars with acids, thereby decreasing the sugar content of squash. These findings further confirm the results of the present study. Similar findings were reported by Šopík et al. (2022) in which quality evaluation for a variety of sugar-based foods was observed at 4 different storage temperatures i.e. -18, 5, 23 and 40 °C for a period of 24 months. They reported that the concentration of glucose, fructose, maltose and sucrose in the food samples decreased at all temperatures during storage, with a more abrupt decrease at the elevated temperature (40 °C).

The present research presented a decline in the total polyphenol content (TPC) and an increasing trend in the total flavonoid content (TFC) of the squash samples during storage. Phenolic compounds are unstable under environmental conditions like temperature, oxygen, pH and light. Oxidative stress can promote the production of ROS (Reactive Oxygen Species) that could degrade phenolic compounds easily. This could be the reason of TPC decline in squash over time. A study was conducted by Mohamad Salin et al. (2022) to determine the possible compositional changes in watermelon juice during its storage at different temperatures. The juice was stored at 4 different temperatures i.e. room temperature, refrigerator cold, refrigerator freeze and freeze dried for a period of 9 days to analyze the physical, chemical and antioxidant properties of the juice. A decline in the polyphenol and flavonoid content was reported which resulted in the deterioration of the juice quality over time. However, the freeze dried retained the maximum nutritional value of the watermelon juice with minimum degradation as compared to other storage temperatures. The rise in TFC in the instant research may be possible due to the breakdown and conversion of complex polyphenols into flavonoids. Polyphenols, through a series of enzymatic reactions, are converted into flavonoids, thereby increasing the TFC content in coconut squash over time. In another research by Purewal et al. (2022), quality analysis of the Kinnow Amla beverages was carried out at refrigerated storage for about 90 days. Different combinations of kinnow and amla juices were mixed for the preparation of the beverage treatments which were then analyzed for their physicochemical and antioxidant properties every fortnight. The outcomes of the study demonstrated a decreasing trend in the TPC of the ready-to-serve beverages, thereby, further confirming the results of the current research.

The antioxidant activity, determined through DPPH (2,2-diphenyl-1-picrylhydrazyl) and FRAP (Ferric reducing antioxidant power) assays, decreased during the storage of the coconut squash at room temperatures. The loss in antioxidant activity can occur due to the decline in total phytochemical content during storage which ultimately resulted in loss of antioxidant activity. These results are in agreement with the findings of Pavlović et al. (2022). They analyzed the phytochemical parameters of beet juice at different storage temperatures for a period of one year. The beet juice was stored in dark at 4 °C, in dark at the room temperature and in light at the room temperature. The antioxidant activity was determined using DPPH assay every month. The storage of the juice at all temperatures resulted in loss of polyphenols which caused a reduction in the total antioxidant activity of the juice. Storage at 4 °C in dark best retained the antioxidant activity with minimal loss of polyphenols as compared to the storage at other temperatures. The findings of Rashid et al. (2021) are in confirmation with the outcomes of the present study. They carried out the physicochemical, phytochemical and sensory analysis of the diet phalsa squash during its storage at room temperature for a period of 90 days. The antioxidant activity of the treatments was determined through FRAP assay. A significant decline in the TPC and antioxidant activity of the treatments was observed. Oxidation of phenolic compounds might be the cause of TPC decline, thereby decreasing the antioxidant activity of the squash over time. A study was carried out by Irshad et al. (2020) in which sea buckthorn squash was prepared and analyzed for its phytochemical profile and antioxidant activity during the storage. For squash preparation, sea buckthorn pulp, sugar syrup, citric acid and sodium benzoate were mixed followed by hydrocolloids addition and stored for 90 days at ambient temperature. The analysis for TPC and DPPH antioxidant activity was carried out at the initiation, 30th, 60th and 90th day of storage. The analysis revealed that the oxidation of polyphenols led to a drop in the total polyphenol content of squash, which ultimately caused a significant loss of antioxidant activity.

In the resent study, the color parameters were observed throughout the storage of squash at room temperature. A drop in the L values was observed throughout the storage intervals which further caused variations in the trend of a*, b*, chroma and hue angle with the storage progression. The decrease in the lightness of the squash treatments in this research may be due to the oxidation reactions promoting the darkness of the squash over time. Also, maillard reaction may cause the browning reactions to accelerate during prolonged storage (Purewal et al., 2022). The use of color retention agents or modified packaging solutions can be incorporated to address the challenges associated with color stability of the drink. Navya et al. (2020) examined the variations in the organoleptic properties of karonda blended squash during its storage period. The squash was prepared by mixing karonda juice with pineapple, guava and papaya juices in different proportions. A decrease in the L values of all the squash treatments was observed at the end of the storage period. Khan et al. (2019) evaluated the quality changes in wood apple and lemon squash over a storage period of 90 days at refrigerated temperature. Quality assessment of the squash revealed an increasing browning index with the increase in the storage days due to non-enzymatic browning reactions between sugars and organic acids. A similar increase in the color intensity i.e. browning index was observed in leh berry squash with the increase in storage days (Selvamuthukumaran & Khanum, 2020).

The free fatty acid (FFA) content plays a vital role as quality indicator of the squash. The FFA content (% lauric acid) increased with the increase in time of storage. The reason for this may be the hydrolysis of fats into free fatty acids, thereby increasing the lauric acid content in the squash. These results are in agreement with the study of Manoj and Shanmugasundaram (2020) in which FFA content in coconut milk increased over time. The findings of Waded et al. (2019) are also in confirmation with the present study in which the physicochemical parameters of coconut spread were studied during its storage at 5 °C. They reported an increase in the FFA content due to the increasing concentration of lauric acid over time.

In the present study, the accelerated shelf-life of the coconut squash was assessed based on the increase in acid value during storage. The squash was stored at the elevated temperature of 40 °C to predict its expected shelf life at room temperature (20 °C). The results showed that T1 had the maximum expected shelf life of 136 days at the room temperature. A similar study was conducted by Hemanth et al. (2020) in which the accelerated shelf life of carbonated guava drink and protein-enriched carbonated guava drink was assessed. The drinks were stored at the elevated temperatures of 14 °C and 40 °C to predict their shelf lives at refrigerated temperature (4 °C) and room temperature (30 °C), respectively. The findings of the study reported that the carbonated guava drink had an expected shelf life of 131 and 14 days at 4 °C and 30 °C, respectively and the protein-enriched carbonated guava drink had an expected shelf life of 60 and 6 days, at the same respective temperatures. In another study conducted by Khasanov and Matveeva (2020), the expected shelf life of a functional beverage was calculated using the Q10 methodology.

Sensory evaluation in the present study was conducted using a 9-point hedonic scale to assess the overall acceptability of the coconut squash treatments. Significant variations were observed for the taste, aroma, aftertaste and overall acceptability of squash, whereas color, flavor, texture and mouth feel showed non-significant variations amongst the treatments. The highest scores for all the sensory parameters were obtained for T5 followed by T4. This means that T5 and T4 had the highest consumer acceptability as compared to other treatments. The increased milk concentration in T5 and T4 enhanced the sensory attributes of the squash making it more preferable and appealing for consumers. T1 had the minimum sensory scores due to its lesser milk content which conferred a diluted taste and lower acceptability amongst the consumers. Similarly, a 9-point hedonic scale was used for the sensory evaluation of the prepared beverages by N. Ahmed et al. (2021), S. R. Moreno et al. (2023) and Thuraisingam et al. (2023) for the assessment of sensory parameters.

## 5 Conclusion

The current research was based on the formulation and accelerated shelf life assessment of coconut squash. Significant variations were observed in the physicochemical and phytochemical parameters of the coconut squash during its storage. T5 was found to be the best treatment due to its superior quality indices. T1 had the maximum expected shelf life at room temperature, followed by T4 and T5. Sensory response by all the participants revealed T5 and T4 to be a preferable choice for all the sensory traits. In a nutshell, T5 was found to be the best formulation paving its way for commercial applications. In the future, studies on the use of natural preservatives to further extend the shelf life of squash while maintaining its sensory and nutritional attributes could be advantageous. Also, the use of color retention agents or modified packaging solutions can be incorporated to address the issues associated with color stability. Furthermore, recipe optimization could improve the shelf life of squash without compromising its quality.

## Ethical statement

Before conducting the sensory evaluation of the analyzed samples, all sensory experts from the Kauser Abdulla Malik School of Life Sciences were consulted and actively involved in the process. The privacy and rights of the experts were fully respected throughout the evaluation. Additionally, all samples used were verified as safe for human consumption. The sensory evaluation committee was duly approved by the Head of the Kauser Abdulla Malik School of Life Sciences.

## Statement of volunteer consent

The sensory experiments conducted in this study were carried out with full respect for the participants' autonomy, ensuring voluntary participation without any form of coercion. Each participant was provided with a comprehensive informed consent form, which included detailed explanations of the study's purpose, procedures, and potential benefits. Participants were given ample opportunity to review the information, ask questions, and seek clarification. Only after fully understanding the details did they sign the informed consent form, indicating their agreement to participate in the study.

## Conflict of interest and authorship conformation form

• All authors have participated in (a) conception and design, or analysis and interpretation of the data; (b) drafting the article or revising it critically for important intellectual content; and (c) approval of the final version.
• This manuscript has not been submitted to, nor is under review at, another journal or other publishing venue.
• The authors have no affiliation with any organization with a direct or indirect financial interest in the subject matter discussed in the manuscript

## CRediT authorship contribution statement

**Sara Shahbaz:** Writing – review & editing, Writing – original draft, Resources, Methodology, Formal analysis, Data curation, Conceptualization. **Iahtisham-Ul-Haq:** Writing – review & editing, Writing – original draft, Visualization, Software, Resources, Data curation, Conceptualization. **Nirmeen Nadeem:** Writing – review & editing, Visualization, Resources, Investigation, Formal analysis, Conceptualization. **Mahnoor Siddiqui:** Conceptualization, Data curation, Methodology, Resources, Software, Writing – review & editing. **Robert Mugabi:** Writing – review & editing, Validation, Software, Methodology, Investigation, Data curation, Conceptualization. **Aanchal Sharma:** Investigation, Methodology, Resources, Software, Validation, Visualization, Writing – review & editing. **Tawfiq Alsulami:** Writing – review & editing, Visualization, Supervision, Resources, Investigation, Funding acquisition. **Gulzar Ahmad Nayik:** Writing – review & editing, Visualization, Supervision, Software, Resources, Methodology, Formal analysis.

## Declaration of competing interest

The authors declare that they have no known competing financial interests or personal relationships that could have appeared to influence the work reported in this paper.

## Data Availability

Data will be made available on request.
